# Effects of the rotavirus vaccine program across age groups in the United States: analysis of national claims data, 2001–2016

**DOI:** 10.1186/s12879-019-3816-7

**Published:** 2019-02-22

**Authors:** Julia M. Baker, Rebecca M. Dahl, Justin Cubilo, Umesh D. Parashar, Benjamin A. Lopman

**Affiliations:** 1 Department of Epidemiology, Rollins School of Public Health, Emory University, 1518 Clifton Road NE, Atlanta, GA 30322 USA; 2 Division of Viral Diseases, National Center for Immunization and Respiratory Diseases, Centers for Disease Control and Prevention, 1600 Clifton Road NE, Atlanta, GA 30333 USA; 3 MAXIMUS Federal, contracting agency to the Division of Viral Diseases, National Center for Immunization and Respiratory Diseases, Centers for Disease Control and Prevention, 1600 Clifton Road NE, Atlanta, GA 30333 USA

**Keywords:** Rotavirus, Gastroenteritis, Vaccination, Hospitalization, United States

## Abstract

**Background:**

The direct effectiveness of infant rotavirus vaccination implemented in 2006 in the United States has been evaluated extensively, however, understanding of population-level vaccine effectiveness (VE) is still incomplete.

**Methods:**

We analyzed time series data on rotavirus gastroenteritis (RVGE) and all-cause acute gastroenteritis (AGE) hospitalization rates in the United States from the MarketScan® Research Databases for July 2001–June 2016. Individuals were grouped into ages 0–4, 5–9, 10–14, 15–24, 25–44, and 45–64 years. Negative binomial regression models were fitted to monthly RVGE and AGE data to estimate the direct, indirect, overall, and total VE.

**Results:**

A total of 9211 RVGE and 726,528 AGE hospitalizations were analyzed. Children 0–4 years of age had the largest declines in RVGE hospitalizations with direct VE of 87% (95% CI: 83, 90%). Substantial indirect effects were observed across age groups and generally declined in each older group. Overall VE against RVGE hospitalizations for all ages combined was 69% (95% CI: 62, 76%). Total VE was highest among young children; a vaccinated child in the post-vaccine era has a 95% reduced risk of RVGE hospitalization compared to a child in the pre-vaccine era**.** We observed higher direct VE in odd post-vaccine years and an opposite pattern for indirect VE.

**Conclusions:**

Vaccine benefits extended to unvaccinated individuals in all age groups, suggesting infants are important drivers of disease transmission across the population. Imperfect disease classification and changing disease incidence may lead to bias in observed direct VE.

**Trial registration:**

Not applicable.

**Electronic supplementary material:**

The online version of this article (10.1186/s12879-019-3816-7) contains supplementary material, which is available to authorized users.

## Background

The United States (US) was one of the first countries to introduce infant rotavirus vaccination nationally [1] and dramatic changes in the rotavirus disease burden and epidemiologic patterns of diarrheal disease have followed [2]. Prior to vaccine introduction in 2006, [3] rotavirus was estimated to cause 55,000–70,000 hospitalizations and over 600,000 emergency room and outpatient/office visits among children under 5 years of age in the US annually [3, 4]. Consistent annual peaks in disease incidence occurred in winter and early spring [4]. Early evaluations of rotavirus seasonality in the post-vaccine era identified substantial alterations of disease patterns, including a reduced magnitude, delayed onset, and shorter duration of the rotavirus season [2]. Further, there has been a distinct shift from annual to biennial peaks in disease incidence among children under 5 years of age, [2, 5, 6] a pattern not observed in some other high-income countries that have introduced the vaccine [7–9].

The direct vaccine effectiveness (VE) of rotavirus vaccine has been evaluated extensively while understanding of indirect vaccine effects is still incomplete. Substantial vaccine impacts are evidenced by 50–90% reductions in rotavirus hospitalizations among young, vaccine-eligible children [10]. A recent meta-analysis estimated a direct VE of 84% against rotavirus-associated hospitalizations or emergency department visits in the US [11]. Notably, this estimate is limited by imperfect rotavirus diagnostics [12] largely due to incomplete testing for rotavirus in the clinical setting [13]. In addition to the remarkable direct effects, reductions in rotavirus disease have exceeded vaccine coverage, suggesting indirect benefits to unvaccinated children [6]. These indirect benefits may extend to children too young to be vaccinated, age-ineligible older children, adolescents, and adults among whom reductions in rotavirus gastroenteritis (RVGE) and all-cause acute gastroenteritis (AGE) have been observed [14].

The long-term impact of a vaccine program will be governed by the direct effects of vaccinating children together with the transmission-modulating consequences of vaccination as described by Halloran et al. [15] Theoretically, direct effects, which represent the biological protection obtained from vaccination at the individual level, [16] are a vaccine characteristic that remains constant over time (except with waning immunity) and are independent of vaccine coverage. Conversely, population-level effects can vary with changes in vaccine coverage, population immunity, and social mixing patterns [15, 17, 18]. These population-level effects include (a) indirect effects or “herd protection” provided to unvaccinated individuals, (b) total effects which describe the combination of biologic and indirect protection received by vaccinated individuals, and (c) the overall effects which quantify the public health benefit of a vaccination program by weighting the total effects among the vaccinated and indirect effects among the unvaccinated populations [15, 19].

Given the relative novelty of the rotavirus vaccine in the US, there have been few evaluations of how vaccine effects may change during the post-vaccine era and their relationship with disease patterns. In order to quantify the full, population-wide impacts of infant rotavirus vaccination, longer-term evaluations of vaccine effects across age groups are needed. Understanding these phenomena could lead to strategies that maximize the program’s benefits and anticipate future healthcare resource needs (e.g. biennial versus annual epidemics). This study aimed to quantify the direct, indirect, overall, and total effectiveness of infant rotavirus vaccination on hospitalization for RVGE and AGE across age groups and their annual variation during the post-vaccine era in the US.

## Methods

### Data source and study period

We analyzed data from the IBM® MarketScan® Commercial Database, a collection of national medical claims and encounters data from commercially insured individuals under 65 years of age in the US. The database includes de-identified, individual-level enrollment, inpatient, and outpatient medical data on employees, their spouses and dependents with employer-sponsored health care insurance in all US states. This encompasses a variety of health plans such as PPOs, POS plans and HMOs but does not include claims covered by Medicaid. The database contains information on several million individuals each year [20].

We analyzed time series data on monthly RVGE and AGE hospitalization rates for July 2001–June 2016. Study years were defined from July through June of the following calendar year and identified by the year in which the rotavirus season occurred (e.g. July 2007–June 2008 was identified as “2008”).

### Identification of RVGE and AGE hospitalizations

Monthly counts of RVGE hospitalizations included individuals with a rotavirus International Classification of Diseases, Ninth and Tenth Revision (ICD-9 and ICD-10) code (008.61 and A08.0, respectively). Given the incomplete detection of rotavirus using ICD coding, [12] data on AGE was also compiled in order to represent possible RVGE not identified as rotavirus-related. Applicable ICD-9/10 codes for AGE include bacterial, parasitic, and viral gastrointestinal illness of determined etiology and presumed infectious or non-infectious gastrointestinal illness of undetermined etiology [21]. For both RVGE and AGE, the ICD-9/10 codes were identified in one of 15 diagnosis fields from inpatient admission claims.

All RVGE and AGE inpatient claims among children, adolescents, and adults not age-eligible to receive the rotavirus vaccine during the study period were included in the analysis and were considered unvaccinated. For children less than 10 years of age who were age-eligible for the vaccine, only those who were continuously enrolled from birth through 6 months of age were included in the analysis. This continuous enrollment requirement aimed to reduce misclassification of vaccination status by helping ensure that rotavirus vaccination occurring within the CDC recommended schedule (at 2, 4 and 6 months of age) [22] was captured in the insurance claim records. Children age-eligible for vaccination but without continuous follow-up were excluded from the analysis because of their unknown vaccination status.

Individuals were grouped into ages 0–4, 5–9, 10–14, 15–24, 25–44, and 45–64 years (data on adults aged 65 years and older are not available in the MarketScan® Commercial Database and were therefore excluded). Children under 5 were additionally categorized into one-year age groups and children under 10 were stratified by vaccination status. The number of enrollment member days were summed by month, year, age group, and vaccination status to provide the enrolled population denominator for each month of the study period and enable calculation of rates.

### Vaccination status

For this analysis, child vaccination status was tracked beginning in July 2006 when the first cohort of newborns became age-eligible for vaccination the following month, coinciding with the Centers for Disease Control and Prevention Advisory Committee on Immunization Practices’ August announcement [3] recommending the vaccine. Children who received at least one dose of either available rotavirus vaccine, RotaTeq (RV5) or Rotarix (RV1) were considered vaccinated. Current Procedural Terminology (CPT) was used to define receipt of RV5 or RV1 based on CPT codes 90680 and 90681, respectively. In order to further reduce misclassification of vaccination status, all individuals (children and adults) residing in states with universal vaccine purchasing programs, which provide immunizations to children free of charge, were excluded from the analysis throughout the study period as vaccination in these states may not be recorded in insurance claim records (Alaska, Connecticut, Idaho, Massachusetts, Maine, North Dakota, New Hampshire, New Mexico, Oregon, Rhode Island, South Dakota, Vermont, Washington, Wisconsin, and Wyoming).

### Statistical analysis

Negative binomial regression models were fitted to monthly RVGE and AGE count data to estimate rate ratios (RR) and 95% confidence intervals (CIs) from which vaccine effects were calculated. Negative binomial models were chosen after overdispersion was identified in preliminary models using Poisson regression. Using the framework proposed by Halloran, [15] we estimated the (a) direct effectiveness of the vaccine by comparing the rates in vaccinated and unvaccinated groups in the post-vaccine era, (b) indirect effectiveness by comparing rates among unvaccinated groups in the post-vaccine era to the pre-vaccine era, (c) overall effectiveness by comparing average rates in the post- and pre-vaccine eras, and (d) total effectiveness by comparing rates in the vaccinated groups in the post-vaccine era to the corresponding groups (all unvaccinated) in the pre-vaccine era. The count of RVGE and AGE cases was modeled using the glm.nb package in R with adjustment for changes in person-years of follow-up using an offset of the log of person-years. Each model included one dichotomous predictor to differentiate the comparison groups: vaccination status (direct), pre- vs. post-vaccine era (indirect and overall), and vaccinated and post-vaccine era vs. pre-vaccine era (total). VE was calculated as 1-RR.

Models were fitted separately for each age group. Direct and total VE was estimated for all age groups eligible for rotavirus vaccination by the end of the study period, with children under 1 year of age vaccine eligible during the entire post-vaccine period (July 2007–June 2016) and children 9 years of age only eligible during the last year of study data (July 2015–June 2016). Indirect and overall VE were estimated for all age groups, though these values were equal for groups ineligible for vaccination throughout the study period because vaccination coverage equaled zero. For the 0–4 age group, direct, indirect, total, and overall effects were additionally calculated for each individual post-vaccine year beginning in 2008 to estimate annual changes in VE. For all age groups, indirect VE was estimated for individual post-vaccine years.

RVGE models were fitted using full-year data. To improve model specificity, AGE models were restricted to the historic rotavirus season of January–June. The year immediately following vaccine introduction, July 2006–June 2007, was excluded as a transition period for all models. The inclusion of a continuous time variable was considered for all models in an effort to adjust for potential secular trends unrelated to vaccination that may have impacted rates. None of the RVGE model results were sensitive to the addition of the time variable based on Akaike information criterion (AIC) and the variable was therefore excluded. Analyses were performed using R software.

### Investigation into annual variation in direct VE

Preliminary estimates of direct vaccine effects among the 0–4 age group appeared to vary annually. Given that direct VE should not change over time (in the absence of waning), we performed calculations to evaluate whether this observed variation could be explained by the combination of imperfect coding of rotavirus and annual variation in disease incidence leading to different magnitudes of misclassification of cases/non-cases in post-vaccine years. In other words, even with constant sensitivity and specificity of ICD-9/10 codes, the number of rotavirus positive/negative cases that are misclassified (and which are ultimately used in calculations of VE) will vary based on the incidence of disease. In years with higher disease incidence, there may be a larger number of individuals misclassified and vice versa. We began with a hypothetical population and applied input parameters of true VE, vaccine coverage, and rotavirus incidence in the unvaccinated population from 2010 to 2016; this enabled estimation of the number of ‘true’ RVGE cases among the vaccinated and unvaccinated children. We then applied realistic values for rotavirus ICD-9 code sensitivity (0.5) and specificity (0.99) [12] to this ‘true’ data to estimate ‘projected’ RVGE cases. From these values, we calculated a ‘projected’ VE and compared this to our ‘observed’ VE estimated via regression analysis and ‘true’ VE (an input parameter).

### Role of the funding source

This study received no dedicated funding. The corresponding author had full access to all data in the study and had final responsibility for the decision to submit for publication.

## Results

From July 2001–June 2016, there were a total of 9211 RVGE hospitalizations and 726,528 AGE hospitalizations across all age groups (Additional file 1: Table S1). Over 91% of RVGE hospitalizations (91.4%) and over half of AGE hospitalizations (54.0%) occurred during the rotavirus season of January–June.

### RVGE time series

#### Young children, 0–9 years of age

**Fig. 1:**
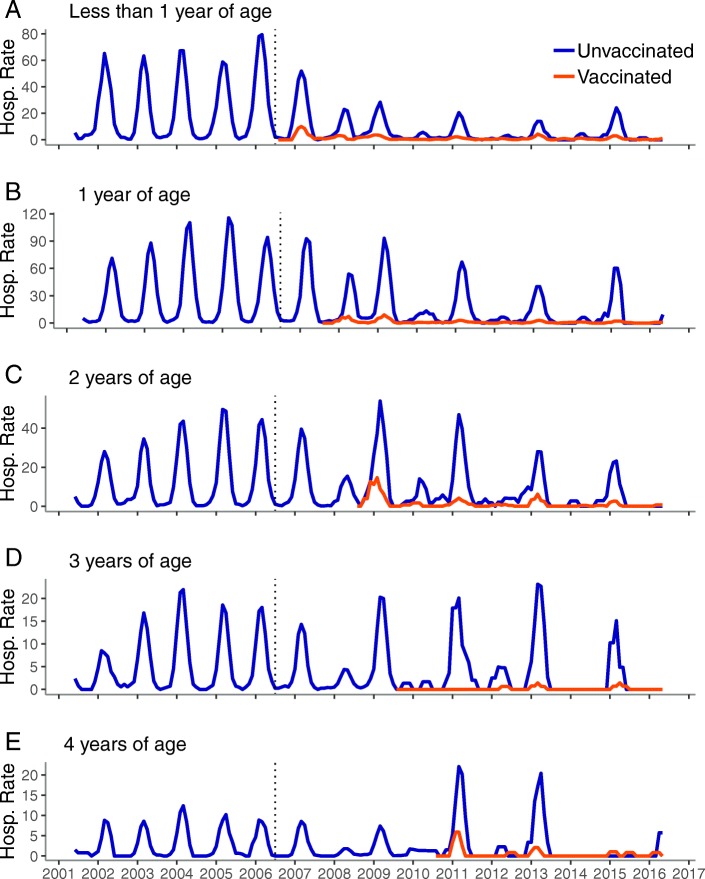
Monthly inpatient RVGE rates per 10,000 person-years, United States, July 2001–June 2016, children 0–4 years.^a^. Legend: **a**: less than 1 year of age, **b**: 1 year of age, **c**: 2 years of age, **d**: 3 years of age, **e**: 4 years of age ^a^ Time series includes all years (including 2007 transition year) and all months (not restricted to the historic rotavirus season), Vertical dashed line represents July 2006 (time of vaccine introduction)

**Fig. 2:**
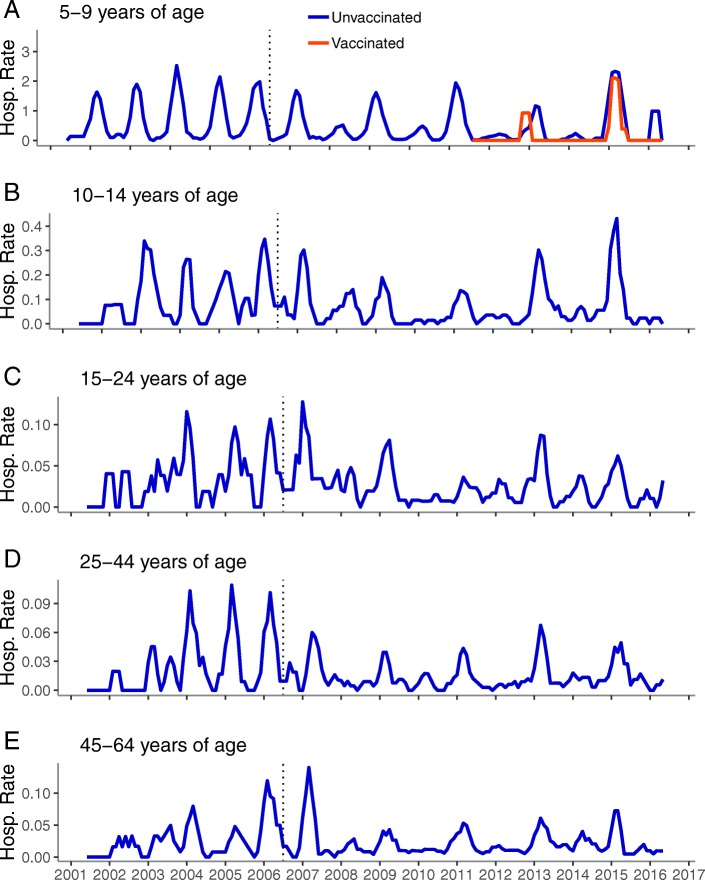
Monthly inpatient RVGE rates per 10,000 person-years by age group, United States, July 2001–June 2016.^a^. Legend: **a**: 5-9 years of age, **b**: 10-14 years of age, **c**: 15-24 years of age, **d**: 25-44 years of age, **e**: 45-64 years of age ^a^ Time series includes all years (including 2007 transition year) and all months (not restricted to the historic rotavirus season). Vertical dashed line represents July 2006 (time of vaccine introduction)

Among children 0–9 years of age, the pre-vaccine period displayed a consistent pattern of rotavirus illness with single annual peaks in hospitalization rates during the winter/spring months (Figs. 1 and 2). Rates declined dramatically for child age groups after introduction of the vaccine and both the 0–4 and 5–9 groups settled into a biennial pattern with the highest rates observed in odd post-vaccine years. When the 0–4 group was further examined by single year and vaccinated/unvaccinated cohorts, the biennial pattern became more apparent among the unvaccinated and younger children. Among the vaccinated children, the biennial pattern was clearest among those 2 years of age and younger. Notably, rates among older, unvaccinated children returned to levels similar to those seen prior to vaccine introduction after initial declines in the post-vaccine period. In contrast, the rates of RVGE remained dramatically lower in the post-vaccine period among vaccinated children.

#### Older children, adolescents and adults

RVGE rates among older children, adolescents, and adults during the pre-vaccine era did not display the same distinctive pattern observed among young children (Fig. 2). Rather, the pre-vaccine period for these groups was characterized by frequent, irregular spikes in rates. After vaccine introduction, the sporadic pattern continued, however, at a lower rate and now punctuated with biennial peaks corresponding to those seen among young children.

### RVGE VE for entire post-vaccine period

The largest declines in RVGE hospitalizations were observed among the youngest children (Table 1). Direct VE was 87% (95% CI: 83, 90%) among children 0–4 years of age.

**Table 1:** Vaccine effectiveness against RVGE hospitalization during the post-vaccine period by age group

Age Group	Direct VE, % (95% CI)	Indirect VE, % (95% CI)	Overall VE, % (95% CI)	Total VE, % (95% CI)
< 1	80* (70, 87)	79* (66, 87)	88* (82, 92)	96* (93, 97)
1	92* (87, 95)	59* (33, 76)	79* (65, 88)	97* (95, 98)
2	87* (76, 93)	43* (4, 67)	68* (44, 83)	93* (86, 96)
3	96* (89, 99)	42 (− 2, 68)	71* (47, 84)	97* (93, 99)
4	81* (53, 93)	36 (− 25, 68)	59* (22, 79)	88* (70, 96)
0–4	87* (83, 90)	60* (48, 69)	78* (71, 83)	95* (93, 96)
5–9	47 (− 12, 79)	48* (30, 61)	50* (34, 63)	72* (42, 89)
10–14		46* (14, 67)	Equivalent to indirect VE^a^	
15–24		42* (10, 62)		
25–44		56* (36, 70)		
45–64		35* (9, 53)		
All ages			69* (62, 76)	

*Represents significance at the alpha = 0.05 level

^a^Indirect and overall VE are equivalent for children, adolescents, and adults over 9 years of age because there are no vaccinated individuals in these age groups

Substantial indirect effects were observed across age groups and these effects generally declined in each older group. Indirect VE against RVGE hospitalization among unvaccinated children under 1 year of age was 79% (95% CI: 66, 87%) compared to adults 45–64 years of age among whom indirect VE was 35% (95% CI: 9, 53%). One exception to this general trend was the greater indirect VE in adults 25–44 years of age (indirect VE: 56%; 95% CI: 36, 70%).

Overall VE against RVGE hospitalizations for the entire study population (all ages) combined was 69% (95% CI: 62, 76%). Significant reductions in hospitalization rates were observed across all ages and the overall vaccine effectiveness generally declined in each older group (Table 1).

Total vaccine effects mirrored the pattern seen in direct and indirect effects, with the largest total VE observed in the youngest children (Table 1, total VE for children 0–4 years of age: 95, 95% CI: 93, 96%).

### RVGE direct and indirect VE by post-vaccine year

**Fig. 3:**
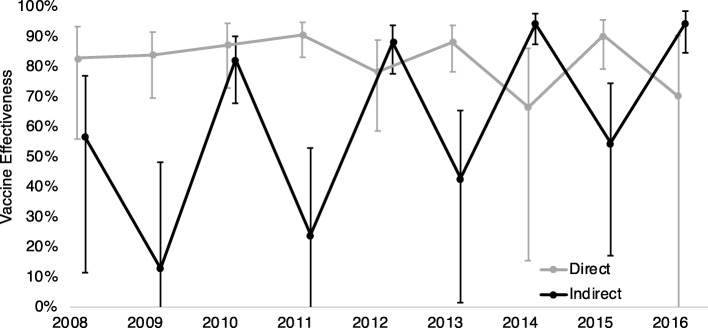
Direct and indirect VE against RVGE by post-vaccine year,^a^ United States, children aged 0–4 years^b^. Legend: ^a^ Post-vaccine years defined as the 12-month period from July through June of the following year. (e.g. “2008” represents July 2007–June 2008). ^b^ VE calculated based on all children in the age group, regardless of age eligibility for rotavirus vaccination, Bars represent 95% confidence limits, Axis truncated at 0%

**Fig. 4:**
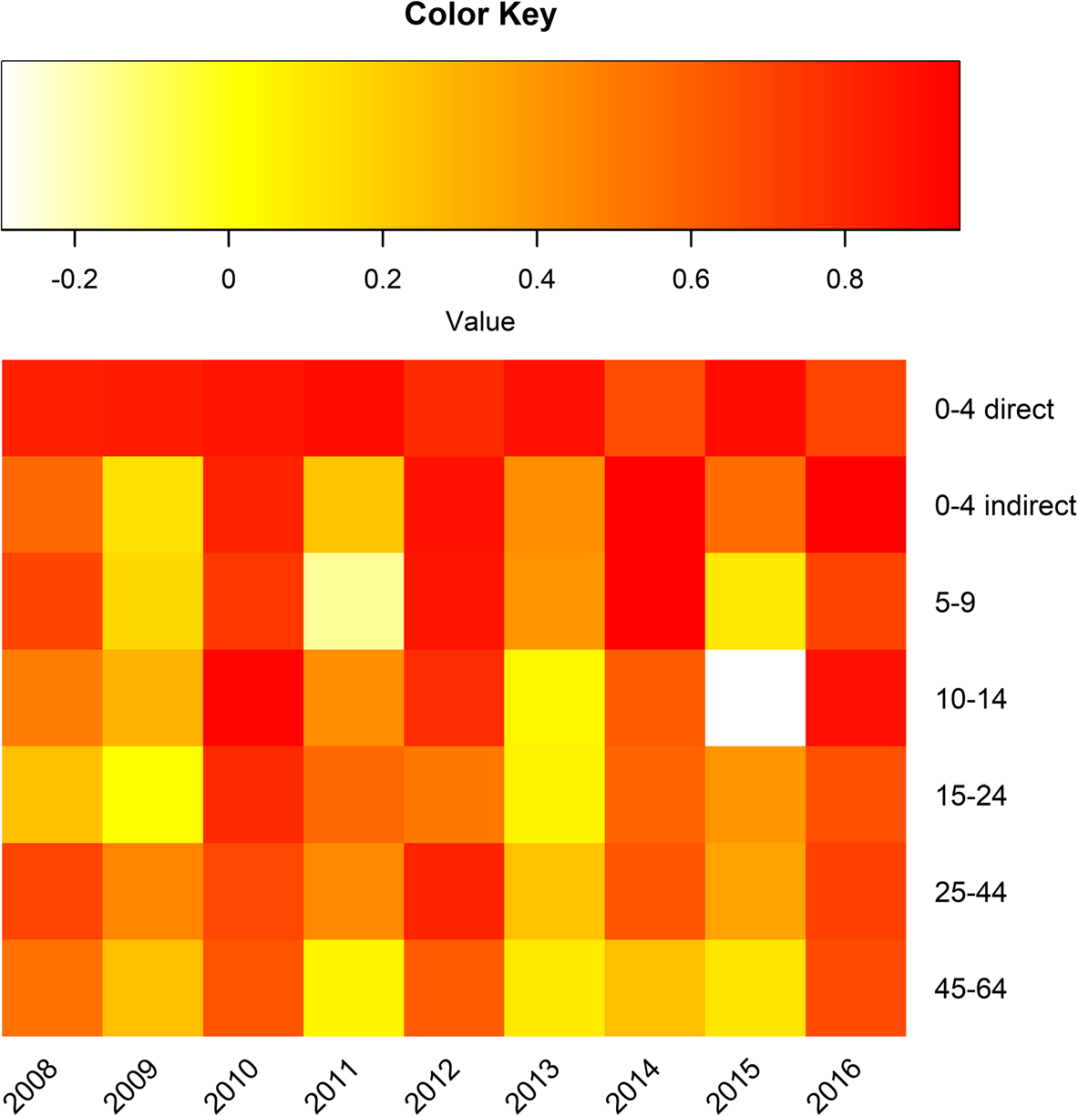
Direct and indirect VE against RVGE for each post-vaccine year,^a^ United States, by age group^b^.  Legend: ^a^ Post-vaccine years defined as the 12-month period from July through June of the following year. (e.g. “2008” represents July 2007–June 2008). ^b^ VE calculated based on all children in the age group, regardless of age eligibility for rotavirus vaccination

After relatively consistent direct VE immediately following vaccine introduction, we observed a possible alternating pattern in estimated direct VE among children 0–4 years of age, with the slightly higher direct VE in odd post-vaccine years (Fig. 3). A more extreme and opposite pattern was apparent for indirect VE for this group, with higher indirect VE during even post-vaccine years compared to odd post-vaccine years. This pattern extended to all ages (Fig. 4). Both VE measures displayed wide CIs due to small numbers of cases.

Calculations to evaluate how variations in disease incidence impacted direct VE revealed a pattern in projected direct VE similar to that of our observed direct VE (Table 2). Our calculations used a population of 1,000,000, vaccine coverage of 50%, true VE of 95%, sensitivity of 0.5, specificity of 0.99, and estimated RVGE incidence among unvaccinated children for each year from 2010 to 2016 from the MarketScan® Commercial Database.

**Table 2:** Projected direct VE calculated in a hypothetical population compared with true and observed direct VE

Direct VE	2010%, (95% CI)	2011%, (95% CI)	2012%, (95% CI)	2013%, (95% CI)	2014%, (95% CI)	2015%, (95% CI)	2016%, (95% CI)
True^a^	95 (90,98)	95 (93, 97)	95 (87, 98)	95 (92, 97)	95 (82, 99)	95 (92, 97)	95 (80, 99)
Observed^b^	87 (73, 94)	91 (83, 95)	78 (59, 89)	88 (78, 94)	67 (15, 86)	90 (79, 96)	70 (−10, 90)
Projected^c^	84 (71, 91)	91 (87, 94)	77 (56, 88)	90 (84, 94)	67 (35, 83)	89 (82, 93)	64 (29, 82)

^a^True VE is a set value used in the calculations

^b^Observed VE is direct VE estimated using the MarketScan® Commercial Database data

^c^Projected VE is the direct VE calculated in a hypothetical population of 1,000,000 with vaccine coverage of 50%, true VE of 95%, sensitivity of 0.5, specificity of 0.99, and estimated RVGE incidence among unvaccinated children based on the MarketScan® data for each year from 2010 to 2016

### AGE time series

**Fig. 5:**
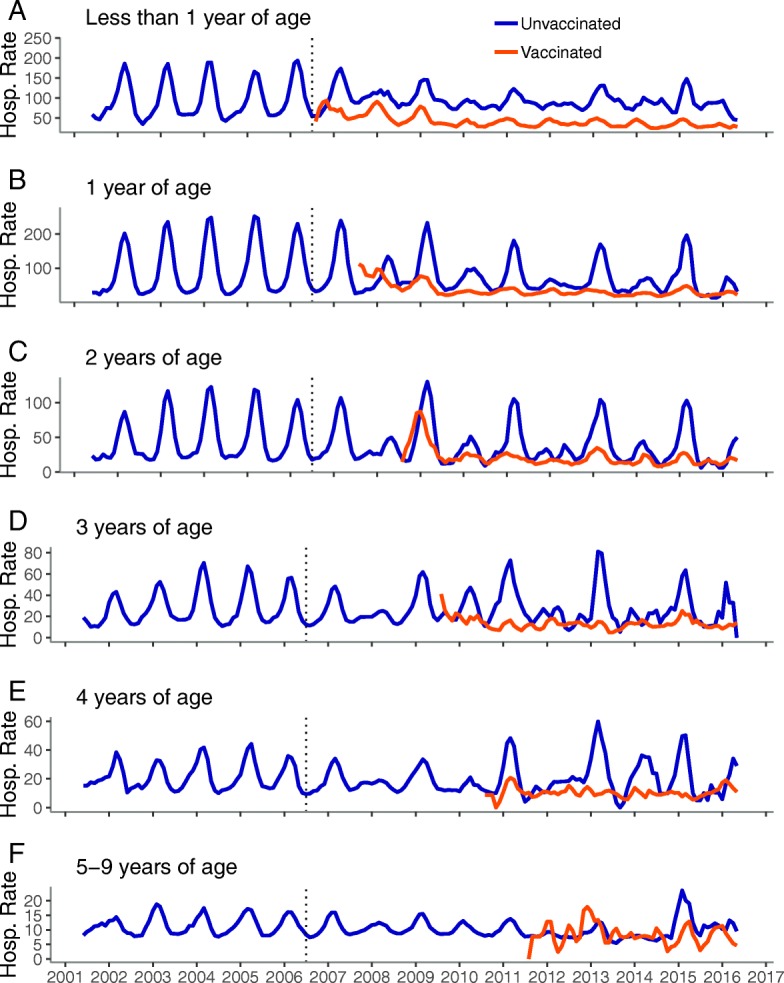
Time series of monthly inpatient AGE rates per 10,000 person-years, United States, July 2001–June 2016.^a^. Legend: **a**: less than 1 year of age, **b**: 1 year of age, **c**: 2 years of age, **d**: 3 years of age, **e**: 4 years of age, **f**: 5-9 years of age ^a^ Timeseries includes all years (including 2007 transition year) and all months (not restricted to the historic rotavirus season). Vertical dashed line represents July 2006 (time of vaccine introduction)

The observations for RVGE rates among children were consistent with those observed for AGE rates in the same population, though the patterns were often less distinct. The pre-vaccine era was characterized by consistent, annual peaks that shifted towards a biennial pattern in the post vaccine era among young children; the biennial patterns were most apparent among the youngest and the unvaccinated children (Fig. 5). Among older children, adolescents, and adults, the pre-vaccine era displayed relatively erratic patterns in AGE rates (Additional file 1: Figure S1). Unlike RVGE rates, the post-vaccine period was not punctuated by clear biennial peaks in these age groups and a slight increasing trend in rates was observed among those age 10 and older.

### AGE VE for entire post-vaccine period

Significant direct, indirect, overall, and total VE was observed among both 0–4 and 5–9 year age groups with the largest impacts among the youngest children (Table 3). Among older age groups, VE estimates were highly sensitive to the method used to control for time. Several methods were considered, including use of a continuous time variable as well as higher order terms. Estimates of VE and their statistical significance varied based on the method used, however, no single model was found to be markedly superior to the rest based on AIC values. Given the uncertainty of these models and output, these results are not presented.

**Table 3:** Vaccine effectiveness against AGE hospitalization during the post-vaccine period by age group

Age Group	Direct VE, % (95% CI)	Indirect VE, % (95% CI)	Overall VE, % (95% CI)	Total VE, % (95% CI)
< 1	59^*^ (54, 64)	25^*^ (12, 36)	49^*^ (37, 58)	69^*^ (63, 74)
1	63^*^ (55, 69)	38^*^ (20, 52)	59^*^ (46, 68)	77^*^ (71, 81)
2	57^*^ (44, 66)	28^*^ (5, 46)	49^*^ (33, 62)	69^*^ (59, 76)
3	60^*^ (49, 68)	16 (− 7, 34)	42^*^ (27, 55)	67^*^ (58, 74)
4	54^*^ (42, 64)	11 (− 13, 29)	33^*^ (16, 46)	60^*^ (47, 70)
0–4	56^*^ (50, 61)	27^*^ (15, 38)	48^*^ (40, 55)	68^*^ (63, 72)
5–9	33^*^ (19, 45)	16^*^ (8, 24)	20^*^ (12, 27)	44^*^ (31, 55)

*Represents significance at the alpha = 0.05 level

## Discussion

Vaccines may have impacts that go beyond their direct, immunological effects. We observed that for rotavirus vaccination in the US, the individual and population-level effects are considerable and complex. First, rotavirus vaccination led to a 95% reduction in RVGE hospitalizations among vaccinated 0–4 year olds. Second, introduction of the vaccine provided 35–60% protection against RVGE hospitalizations to unvaccinated individuals across age groups; this protection was generally limited to even post-vaccine years. Parallel patterns in indirect effects observed across all ages highlight the underrecognized burden of rotavirus outside the pediatric age range and emphasize the importance of infants in disease transmission. Lastly and surprisingly, estimates of direct VE varied annually, but we demonstrated that this observation is consistent with biases resulting from ICD-9/10 misclassification combined with biennial incidence patterns rather than variable vaccine performance.

The decade-long post-vaccine period in the US provides a unique opportunity to assess the longer-term impacts of rotavirus vaccination across age groups and to quantify specific vaccine effects. A central strength of this study was the compilation and analysis of 15 years of national data from the MarketScan® Commercial Database. The large size, comprehensive information on vaccination status and consistent coding [20] enabled detailed analysis of nine years of post-vaccine data including age- and year-specific vaccine effects. Previous studies assessing the impacts of the rotavirus vaccine are limited to short-term post-vaccine periods, limited geographic ranges, or pediatric age groups [14, 19, 23, 24]. This study contributes to existing literature on the effects of rotavirus vaccination across the age range [25] and is the first to estimate annual variation in vaccine effects over the nearly decade long post-vaccine period.

Overall, among all age groups combined, rotavirus hospitalization rates declined by nearly 70% after introduction of the vaccine. The youngest children were impacted most, however, the effects of the vaccine program were also felt outside the pediatric age range, bolstering existing evidence of indirect vaccine effects in the more immediate period following vaccine introduction [11, 25]. Population-wide indirect benefits of infant vaccination were demonstrated by reductions in RVGE hospitalization rates across age groups coupled with the emergence of biennial peaks in rates corresponding to those seen among vaccinated children. These findings reinforce the notion that infants are primary drivers of rotavirus infection across age groups. This theory is further supported by the increase in indirect benefits suggested among adults 25–44 years of age, a population likely to have close contact with young children [26]. Yet, the general decline in indirect vaccine effects in older age groups indicate that rotavirus infection among these unvaccinated populations are not solely driven by infants.

The biennial pattern in disease incidence may have influenced our estimation of direct vaccine effectiveness. Imperfect classification results in a bias in vaccine effectiveness; if incidence is changing, the magnitude of the bias will vary because the number of cases to which the sensitivity and specificity of the codes are being applied changes. Indeed, we found that the annual variation in observed direct VE is entirely consistent with a vaccine with constant true effectiveness, imperfect sensitivity/specificity of hospital coding, and varying incidence. In other words, variation in direct effectiveness may be due to the biennial patterns in disease incidence rather than true changes in vaccine effects. This bias could potentially arise in other estimates of direct vaccine effectiveness measured in the context of varying disease incidence and imperfect disease classification.

Cycles are a well-documented [11] feature of many infectious diseases. When host immunity combines with some seasonal factor (e.g. school terms or weather) season cycles may emerge [27]. Vaccination, which serves to reduce the number of individuals susceptible, may perturb these patterns and increase the inter-epidemic cycle. Indeed, this was predicted to occur for rotavirus under some epidemiological and vaccine-coverage scenarios [28–30]. Our study adds to the empirical data supporting this idea, but also extends it by documenting that these effects ripple across the age range. During even (low incidence) years, indirect VE was high across the age range while in odd (high incidence) years, there was little-to-no indirect VE.

Important limitations should be noted. We relied on ICD-9/10 codes which imperfectly capture RVGE. Not all individuals hospitalized for AGE are tested for rotavirus; evaluations among children during the pre-vaccine era have demonstrated that rotavirus ICD-9 coding has high specificity (97%) and low sensitivity (less than 50%) [12]. Little is known about the specificity and sensitivity of the coding in the post-vaccine era, possible misclassification or incomplete coding in the MarketScan® Commercial Database, frequency of testing among adults, [31] or temporal changes in testing practices since vaccine introduction. One approach to address this limitation was to assess disease patterns in AGE rates, which have been shown to be valuable in assessing the burden of severe RVGE [13]. While we observed consistent patterns in RVGE and AGE in young age groups, vaccine impacts were not clear in older age groups, perhaps because an effect was overwhelmed by an increasing secular trend in AGE among older individuals (see additional materials).

A primary concern in analysis of time series data is time varying confounders. We aimed to adjust for potential unknown temporal trends by testing the sensitivity of all models to the inclusion of a sequential time variable; none of the RVGE models were found to be sensitive to this variable. National coverage levels for the rotavirus vaccine increased in the years immediately following its introduction though have plateaued around 73% since 2013 [11, 32]; coverage may indirectly contribute to the variation in direct and indirect effectiveness observed by impacting the number of susceptibles in the population. There is evidence of changes in prevalence of circulating rotavirus strains in the US since vaccine introduction though no consistent pattern has been observed [33], making this unlikely to be the driver of the distinct patterns observed for RVGE rates and VE. It is possible that increased frequency of testing [34] and improved laboratory techniques [35] may impact the number of RVGE cases over the post-vaccine period. If these changes have occurred, they would likely result in an underestimation of the VE measures. Finally, this study may have limited generalizability as the data used did not include the under-insured, individuals on Medicaid, and individuals aged 65 years and older. We are unable to draw conclusions about specific patterns of illness or VE among populations not included in the dataset, such as the underinsured who may have different levels of vaccine coverage [36]. Nonetheless, the effects observed are a function of the wider US population, not just those captured in the dataset.

This study provides new evidence of the individual and population-wide impacts of the rotavirus vaccine and highlights an important potential for bias in direct VE estimation, not previously investigated for rotavirus vaccination. Measurements of direct rotavirus VE may be prone to downward bias in the post-vaccine era due to reductions in disease incidence resulting in lower and changing predictive value of diagnosis. A vaccinated child in the post-vaccine era has a 95% reduced risk of RVGE hospitalization compared to a child in the pre-vaccine era. Vaccine benefits extended to unvaccinated individuals across the age range and demonstrate the important role of infants in rotavirus transmission.

## Conclusions

This comprehensive estimation of the range of vaccine effects provides new evidence of the individual and population-wide impacts of infant rotavirus vaccination and highlights an important potential for bias in direct vaccine effectiveness estimation. Our findings demonstrate the high direct effectiveness of infant rotavirus vaccination and suggest that the impacts of the vaccine program can be felt population-wide, including among adults and unvaccinated children. A novel finding was that imperfect disease classification combined with changing disease incidence during the post-vaccine period may lead to downward bias in the estimated direct vaccine effectiveness. This bias should be considered in other estimates of direct vaccine effectiveness in the context of varying disease incidence and imperfect case classification.

## Additional file

Additional file 1: **Table S1.** Number of RVGE and AGE cases by age group, United States, July 2001–June 2016.^a^. **Figure S1.** Time series of monthly inpatient AGE rates per 10,000 person-years by age group, United States, July 2001–June 2016.^a^. (DOCX 20584 kb)

## Abbreviations

AGE: All-cause acute gastroenteritis
AIC: Akaike information criterion
CI: Confidence interval
CPT: Current Procedural Terminology
ICD-10: International Classification of Diseases, Tenth Revision
ICD-9: International Classification of Diseases, Ninth Revision
RR: Rate ratio
RV1: Rotarix
RV5: RotaTeq
RVGE: Rotavirus gastroenteritis
US: United States
VE: Vaccine effectiveness
