# Supplementary material for: Effects of the rotavirus vaccine program across age groups in the United States: analysis of national claims data, 2001–2016
Source: BMC Infect Dis. 2019 Feb 22;19:186. doi: 10.1186/s12879-019-3816-7 (PMC6387516; doi:10.1186/s12879-019-3816-7)
Supplement: Supplementary file 1 — Table S1. Number of RVGE and AGE cases by age group, United States, July 2001–June 2016.a. Figure S1. Time series of monthly inpatient AGE rates per 10,000 person-years by age group, United States, July 2001–June 2016.a. (DOCX 20584 kb) [file 12879_2019_3816_MOESM1_ESM.docx]

**ADDITIONAL MATERIAL**

Table 1. Number of RVGE and AGE cases by age group, United States, July 2001- June 2016.^a^

| **Age Group** | **RVGE** | **AGE** |
| --- | --- | --- |
| <1 | 1,908 | 15,647 |
| 1 | 2,879 | 11,959 |
| 2 | 1,545 | 6,857 |
| 3 | 689 | 4,624 |
| 4 | 477 | 4,169 |
| 0-4 | 7,498 | 43,256 |
| 5-9 | 865 | 18,469 |
| 10-14 | 206 | 18,474 |
| 15-24 | 152 | 64,326 |
| 25-44 | 190 | 189,900 |
| 45-64 | 300 | 392,103 |
| All ages | 9,211 | 726,528 |

^a^ Includes all years (including 2007 transition year) and all months (not restricted to the historic rotavirus season)

Figure 1. Time series of monthly inpatient AGE rates per 10,000 person-years by age group, United States, July 2001- June 2016.^a^


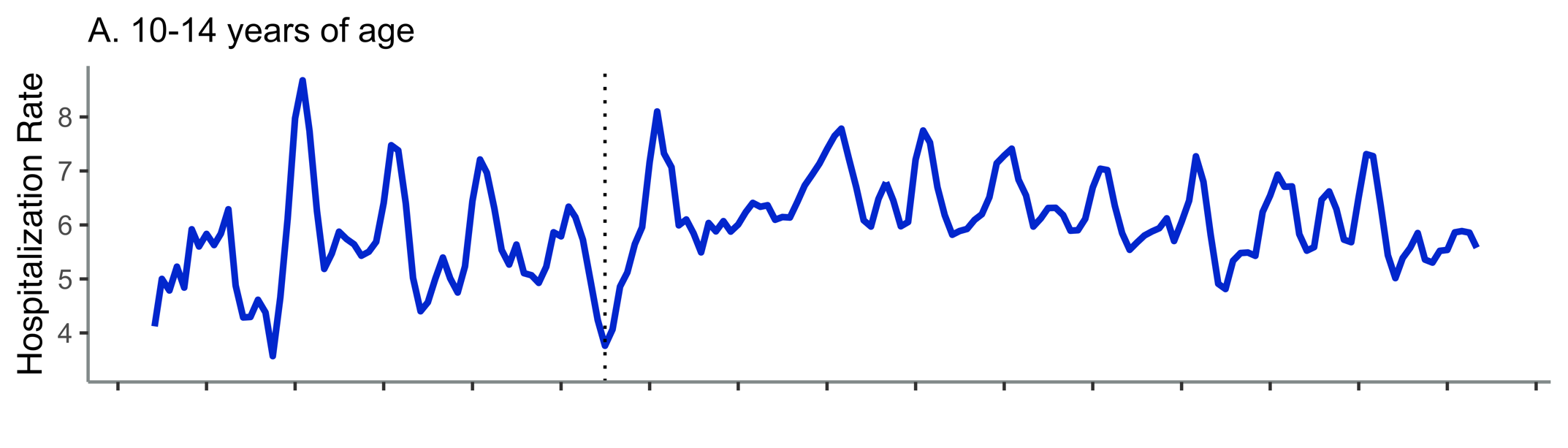


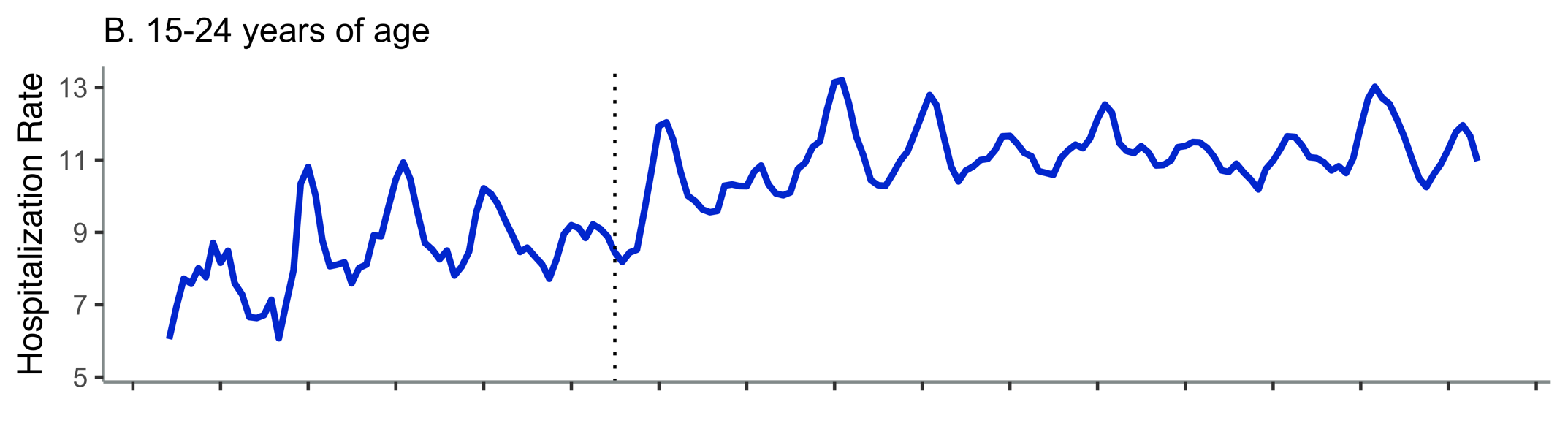


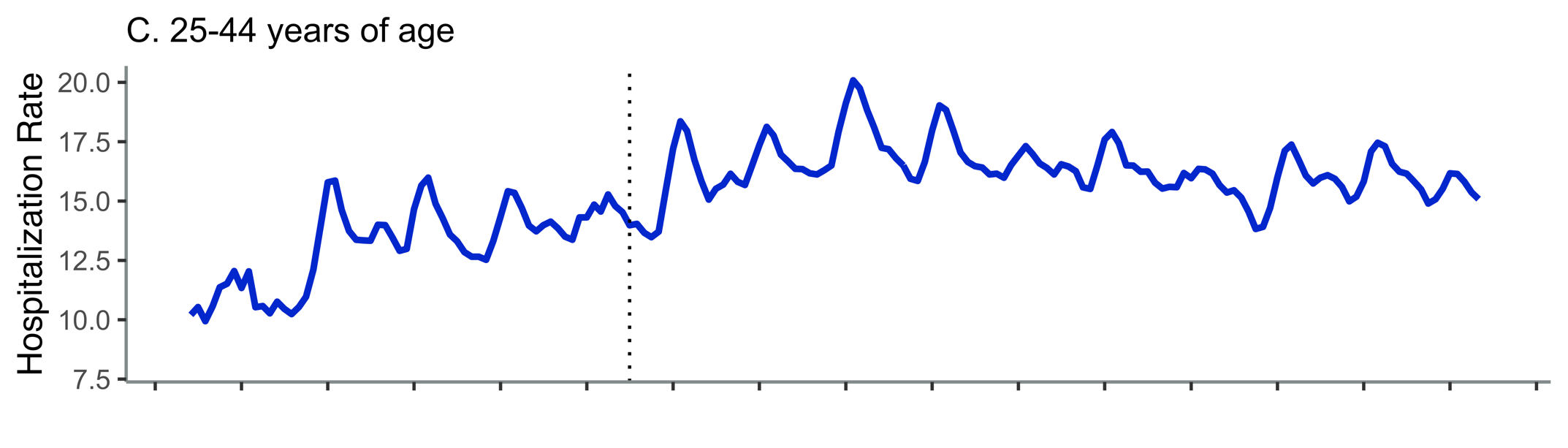


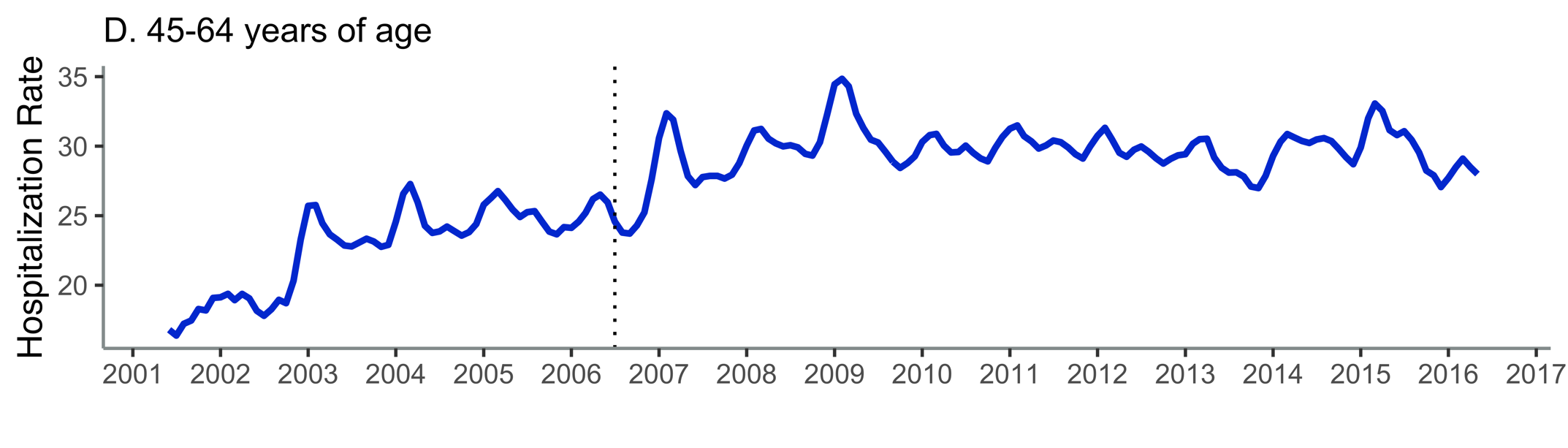


^a^ Timeseries includes all years (including 2007 transition year) and all months (not restricted to the historic rotavirus season)

Vertical dashed line represents July 2006 (time of vaccine introduction)
